# Medical Colleges

**Published:** 1919-04

**Authors:** 


### Medical Colleges.

The pre-medical schools of the country will be closing the last of May and in June. The students will then be selecting their medical colleges and will be consulting established physicians as to the best place to go. Watch the advertising columns of The Texas Medical Journal, especially in June, for announcements from a number of the best colleges that offer the strongest inducements to medical students.

